# Modes of Tubercular Infection in Wild Animals in Captivity

**Published:** 1903-05

**Authors:** W. Reid Blair

**Affiliations:** Resident Veterinarian and Pathologist, New York Zoological Park


### MODES OF TUBERCULAR INFECTION IN WILD ANIMALS IN CAPTIVITY.

By W. Reid Blair, D.V.S.,

RESIDENT VETERINARIAN AND PATHOLOGIST, NEW YORK ZOOLOGICAL PARK.

In the absence of the more positive information which one acquires from a long series of experiments designed for the purpose of ascertaining the priority and manner of invasion of tuberculosis, much of this information regarding the progress of the lesions has been gathered from post-mortem examinations of natural cases. This is particularly the case in animals whose price has prevented them from figuring largely in experimental pathology.

As the existence of tuberculosis is determined by the presence of the tubercle bacillus, which produces the disease, consequently it is only since the characters of this were made known that we have been able to make an absolute diagnosis in suspected cases.

The identity of tuberculosis in human beings and that of certain animals, and the possibility of one infecting the other, renders this disease of the greatest importance.

The great difficulty in determining when the animal first becomes tuberculous makes it practically impossible to prevent the possibility of infection to its companions. Particularly is this danger greater among primates, where it is necessary to confine from six to ten, or even more, in one cage.

*Animals Affected.* While it is quite safe to say that hardly any animal possesses absolute immunity from tuberculosis, certain species and individuals are undoubtedly less susceptible than others.

My investigations have from necessity been confined to the animals in the primate collection, owing to the fact that with one or two exceptions the animals in the park outside the primates have been free from this disease. The experimental work along this line is not complete, but the facts already gathered are of importance.

The examinations were conducted as follows: As soon after death as possible the animal was opened; the trachea from larynx to bifurcation was ligatured at each end and removed. Smears and scrapings were then taken, under sterile conditions, from mucous membrane, five to six slides used in each instance. A

like number of specimens were taken from the nostrils, under the same conditions, at the same time.

Smears were taken from the living animal by means of small cotton swabs applied to the mucous membrane of the throat or nostrils.

Smears taken from the nostrils of suspected cases and those that showed no clinical signs whatever were interesting in demonstrating that at one time the bacilli were present in great numbers, while at other times (intervals of one or two weeks) we find them few in number or wholly absent in the same animal; hence it would seem that too great reliance cannot be placed on the occurrence of bacilli in the nostrils as indicating a diseased animal, for in several instances bacilli were found in the secretions from the nostrils when on careful autopsy no evidences of tuberculosis were found. The bacilli were found to be fairly constant in advanced cases of pulmonary lesions where breaking down of tissue was a distinct feature. Coughing is rarely present among these animals, even in the most advanced cases, but sneezing is quite frequent, even in health, and this, it seems to me, is the most prolific source of dissemination of the contagium. Since the bacilli when dried may be carried by currents of air, it is not necessary that healthy animals should come in direct contact with the tuberculous cases to become infected.

Without the *bacillus tuberculosis* the disease cannot be contracted even by the most weakly animal; but it is equally true that, with its presence in a building or in the body of a companion, the strongest is not absolutely free from the danger of contagion. Notwithstanding the frequency of extensive pulmonary lesion, the trachea, larynx, and pharynx are seldom affected with tuberculosis in these animals. I found lesions in the larynx in only one instance, but in three cases discovered an occasional bacillus within the epithelial cells lining this organ. Some appeared to be in process of degeneration. The bacilli were never in sufficient numbers to give rise to any distinct lesions. Two of these cases had no lesions of tuberculosis present in any part of the body on examination. This fact would seem to indicate that the lining cells of trachea and larynx possess considerable phagocytic power.

*Primary Infection by Inhalation.* In a large percentage of the cases examined, the lungs with their lymph glands (especially the nodes situated at bifurcation of trachea) showed calcareous deposits, while other lymphatic nodes were œdematous, or in process of caseation. I am led to believe that primary infection takes place in the great majority of cases by way of the respiratory tract. It seems to me probable that tubercle bacilli enter the lungs and pass to communicating glands without giving rise to preliminary lesions of the organ with which they first come in contact.

Of the smears taken from different parts of the larynx and trachea where pulmonary tuberculosis existed in over 90 per cent. of the cases, tubercle bacilli were found in all parts of the tube.

In a small number of cases tubercle bacilli could not be found in the trachea, though the lungs showed far-advanced tuberculosis, the tubercles showing calcareous degeneration. In one instance (that of a small macaque monkey) one lung was totally functionless, appearing as a large, calcareous mass attached firmly to the costal pleura; the other being only moderately affected. Yet the animal was apparently well nourished, as evidenced by the amount of flesh and fat present. In this case I was unable to demonstrate the bacillus in the trachea.

An interesting case was that of a spotted lemur which was slightly injured, necessitating its isolation, temporarily, in the hospital room. This animal presented a fairly healthy appearance (excepting for the injury), with no clinical symptoms whatever which led me to have the slightest suspicion that the animal was tuberculous. Six smears were taken from throat and nostrils, all of which showed tubercle bacilli in abundance, those of the throat being particularly numerous. This animal was never again put on exhibition, and I did not have to wait long to confirm my diagnosis, as the animal died within a few days. The autopsy showed far-advanced pulmonary, pleural, and pericardial tuberculosis. No lesions were present in other organs.

*Infection by Ingestion.* While one must take into consideration the possibility of primary invasion taking place by the intestinal canal through the bacilli taken in with food or contaminated drinking-water, this, in my opinion, is not the common source of infection, but that intestines and abdominal organs are usually infected secondarily, through the breaking down of tubercular deposits in the lungs, finding their way into the bronchial tubes, finally reaching the throat, the animal swallowing secretion containing the bacilli in great numbers, some of which would doubtless escape the action of the gastric juices, pass on to the intestines, and, if in sufficient number, produce tubercular enteritis, or they might pass to mesenteric glands without producing any lesions whatever in the intestines.

Experimental evidence apparently shows that a relatively large number of bacilli are necessary to experimentally infect healthy animals by ingestion. Probably if the mucous membrane be not intact a smaller number of the bacilli would suffice.

The rarity or total absence of tubercular lesions in the stomach would indicate that the gastric juices possess power to prevent the growth of the bacilli.

Specimen smears were taken from the œsophagus at the middle and lower third. Although I have made numerous smears, I have in only a few instances found the bacilli to be in great numbers, and in a large percentage of cases none was present.

The method used in staining was that of Gabbett. After spreading the material in the finest possible film upon the glass slide, a fluid composed of 100 grammes of a 5 per cent. aqueous solution of carbolic acid and 10 grammes of absolute alcohol in which 1 gramme of carbol-fuchsin had been dissolved. A few drops of this solution was poured over the film side of the slide and heated for two minutes, or until steam arose from the stain. It was then placed for about one minute in a mixture of 100 grammes of a 2 per cent. solution of sulphuric acid in which 2 grammes of methylene-blue had been dissolved. It was next rinsed in alcohol and mounted in Canada balsam; microscopic examination with a 1/12 oil-immersion lens used.

By this convenient method the bacilli appear red or pink and the surrounding tissue blue or greenish in color.

The fact that we are not at the present time seriously affected with tuberculosis is no reason why we should not take every possible precaution to prevent and to repress its further advance.

